# Realist evaluation to improve health systems responsiveness to neglected health needs of vulnerable groups in Ghana and Vietnam: Study protocol

**DOI:** 10.1371/journal.pone.0245755

**Published:** 2021-01-22

**Authors:** Tolib Mirzoev, Ana Manzano, Bui Thi Thu Ha, Irene Akua Agyepong, Do Thi Hanh Trang, Anthony Danso-Appiah, Le Minh Thi, Mary Eyram Ashinyo, Le Thi Vui, Leveana Gyimah, Nguyen Thai Quynh Chi, Lucy Yevoo, Doan Thi Thuy Duong, Elizabeth Awini, Joseph Paul Hicks, Anna Cronin de Chavez, Sumit Kane

**Affiliations:** 1 Nuffield Centre for International Health and Development, University of Leeds, Leeds, United Kingdom; 2 School of Sociology and Social Policy, University of Leeds, Leeds, United Kingdom; 3 Department of Population and Reproductive Health, Hanoi University of Public Health, Hanoi, Vietnam; 4 Research and Development Division, Ghana Health Service, Accra, Ghana; 5 Department of Undergraduate Education, Hanoi University of Public Health, Hanoi, Vietnam; 6 School of Public Health, University of Ghana, Accra, Ghana; 7 Department of Quality Assurance, Institutional Care Directorate, Ghana Health Service, Accra, Ghana; 8 Mental Health Authority, Accra, Ghana; 9 Nossal Institute for Global Health, University of Melbourne, Melbourne, VIC, Australia; University of Cape Coast, GHANA

## Abstract

**Background:**

Socio-economic growth in many low and middle-income countries has resulted in more available, though not equitably accessible, healthcare. Such growth has also increased demands from citizens for their health systems to be more responsive to their needs. This paper shares a protocol for the RESPONSE study which aims to understand, co-produce, implement and evaluate context-sensitive interventions to improve health systems responsiveness to health needs of vulnerable groups in Ghana and Vietnam.

**Methods:**

We will use a realist mixed-methods theory-driven case study design, combining quantitative (household survey, secondary analysis of facility data) and qualitative (in-depth interviews, focus groups, observations and document and literature review) methods. Data will be analysed retroductively. The study will comprise three Phases. In Phase 1, we will understand actors’ expectations of responsive health systems, identify key priorities for interventions, and using evidence from a realist synthesis we will develop an initial theory and generate a baseline data. In Phase 2, we will co-produce jointly with key actors, the context-sensitive interventions to improve health systems responsiveness. The interventions will seek to improve internal (i.e. intra-system) and external (i.e. people-systems) interactions through participatory workshops. In Phase 3, we will implement and evaluate the interventions by testing and refining our initial theory through comparing the intended design to the interventions’ actual performance.

**Discussion:**

The study’s key outcomes will be: (1) improved health systems responsiveness, contributing to improved health services and ultimately health outcomes in Ghana and Vietnam and (2) an empirically-grounded and theoretically-informed model of complex contexts-mechanisms-outcomes relations, together with transferable best practices for scalability and generalisability. Decision-makers across different levels will be engaged throughout. Capacity strengthening will be underpinned by in-depth understanding of capacity needs and assets of each partner team, and will aim to strengthen individual, organisational and system level capacities.

## Introduction

Responsiveness is a key goal of any national health system, and can be defined as “…when institutions… are cognisant and respond appropriately to the universally legitimate expectations of individuals… safeguarding of rights of patients to adequate… care” [1 p.3]. In addition to being an intrinsic goal, responsiveness is also a process involving multiple interactions within health systems [2] and is an integral value within health systems [3].

**Fig 1:**
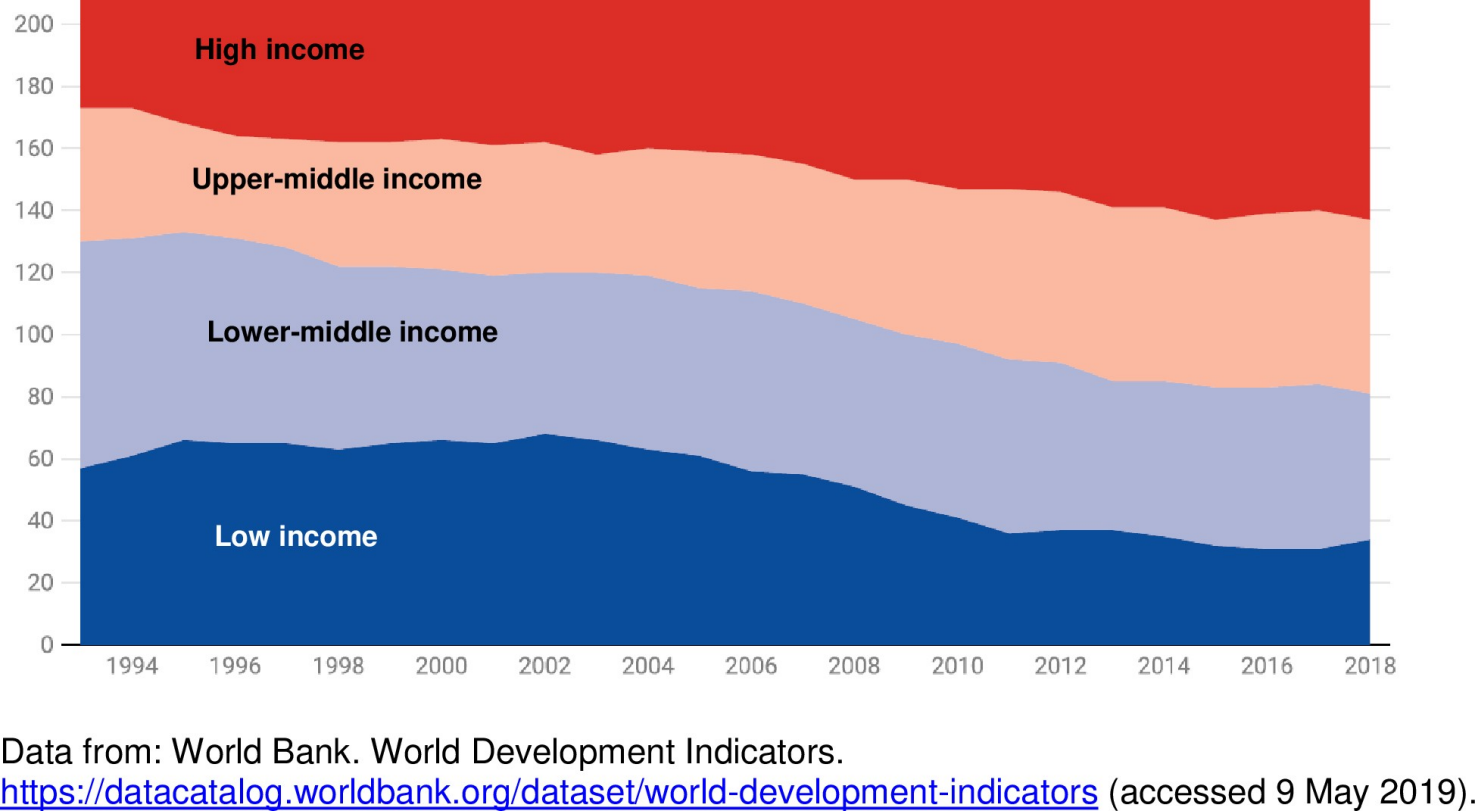
Number of countries by income category, over time.

In the last two decades, large parts of the world have seen significant and rapid economic growth [4]. Between 2003 and 2018, 32 of 66 low-income countries have become middle-income (Fig 1), and today the vast majority (73%) of people live in middle-income countries. Such growth has led to major improvements in availability of healthcare in those contexts. Citizens are now no longer satisfied with mere availability of services but increasingly demand systems to be responsive to their medical and non-medical social expectations, for example treatment with dignity and confidentiality [1, 5].

Improved responsiveness, while itself being a health systems goal, also contributes towards equitable improvements in population health. Responsive systems can improve service uptake, ensure adherence to treatment, and ultimately enhance patient welfare [6, 7]. People, especially the most vulnerable, are more likely to use services if health systems are responsive to their expectations. Conversely, people are unlikely to use services within unresponsive systems [5, 7, 8]. Yet of the four intrinsic goals of health systems i.e. improving health; ensuring fairness in financing, efficiency and responsiveness [9, 10], responsiveness is the least studied, particularly in low and middle-income countries (LMICs) [2, 11].

Improving health systems responsiveness requires targeting internal interactions (between healthcare staff and managers within health systems) and external interactions (between people and healthcare staff) [2]. Both interactions require, and in turn contribute towards, strengthening of different health systems components. This includes staff support and development, information exchange, communication and service delivery.

Vulnerability, defined as defencelessness, insecurity and exposure to risks [12], comprises two elements: exposure to risks (financial, physical, psychological) and inability to deal with risks without suffering further damage. Anyone can become vulnerable, but some groups in certain contexts such as pregnant women, children and the elderly are often considered as the most vulnerable in society [13].

Vulnerability of pregnant women is a particularly complex and contested issue [14]. Scholars cautioned against unnecessary medicalisation of pregnancies from the feminist position and sociology of health [15], highlighting a vicious cycle of exclusion of pregnant women from biomedical research leading to them losing out on potentially safe treatments [16] and raising vulnerability as a relational and context-specific issue which needs safeguarding within research studies [17].

Despite significant progress, improvements in maternal health remain highly inequitable in many LMICs. This includes Ghana and Vietnam where large disparities cut across income, ethnicity, residence and other aspects of intersectionality [18, 19] and there is limited utilisation of healthcare by the most vulnerable. For example, in Vietnam women from ethnic minorities have 30% fewer antenatal care visits and facility deliveries [19]. Yet, despite the vulnerable having major needs [20], their voices are often neglected in health systems [21].

Psychological wellbeing in pregnancy and postpartum is crucial to mother’s and child’s health [22]. However, mental health is often the most neglected aspect of maternal health [20, 23]. A truly responsive health system should recognise the intersectional nature of vulnerability and effectively identify and respond to the neglected health needs of vulnerable groups, such as maternal mental health. It is particularly important within the context of LMICs, where power differentials between health providers and service users are often heightened due to substantial information asymmetry between these groups, which contributes to perceptions of risks in pregnancy being most shaped by healthcare providers [17].

This paper shares a protocol for the RESPONSE study which aims to understand and improve health systems responsiveness to health needs of vulnerable groups in Ghana and Vietnam. RESPONSE seeks to contribute to advanced understanding and improving health systems responsiveness in LMICs through multiple case studies in Ghana and Vietnam. We will co-produce with service providers and service users, implement and evaluate context-sensitive interventions to improve systems responsiveness. The focus of our study is on improving health systems responsiveness to the needs of pregnant women, particularly those with mental health problems. Pregnant women were chosen as a priority group who meets the criteria for earlier-discussed vulnerability, due to the intersection of their gender, age, ethnicity, socio-economic status, health needs and other characteristics of intersectionality.

## Methods

### Study objectives and questions

Our overall health systems research question is: In what way can health systems become more responsive to neglected health needs of vulnerable groups within the contexts of lower-middle-income countries? This study has five objectives, shown in Table 1 alongside the corresponding research questions.

**Table 1:** Study objectives and research questions.

Study objectives	Specific research questions
1) Conduct in-depth analyses of how health systems responsiveness is understood and enacted by key health systems actors, and to what degree the local health systems are responsive to these expectations	1. How is health systems responsiveness understood and enacted by key health systems actors in LMICs? a. What do people, especially from vulnerable groups, expect from a responsive system and how do these shape their engagements with their health systems? b.What being responsive means to key actors on the ‘systems’ side (providers, managers, policymakers), and how do these shape their practices? 2. To what degree the local health systems organise themselves to be responsive to actors' expectations? a. How do systems contexts, structures and processes facilitate or constrain its responsiveness?
2) Co-produce, implement and evaluate context-sensitive interventions to improve health systems responsiveness to neglected health needs of vulnerable groups	
3) Develop an empirically-based and theoretically-grounded model of complex relations between the contexts, the mechanisms and the outcomes of the interventions to improve health systems responsiveness	3. Which co-produced interventions can improve health systems responsiveness to neglected health needs of vulnerable groups? a. How does improving responsiveness affect the use of mental and maternal healthcare? b. In what way responsiveness depends on, and contributes to, other health systems goals?
4) Develop transferable best practices for scalability and generalisability of the pilot-tested interventions	4. Which transferable best practices can be developed for future health systems strengthening? a. What adaptations will be required to apply the interventions to other health areas? b. In what way can the model be adapted to improve responsiveness in other contexts?
5) Strengthen research capacity at individual, organisational and systems levels, through extending existing collaborations into strong South-South and South-North exchange and learning within and between Ghana, Vietnam and UK	

Key study’s outcomes will be: (1) improved health systems responsiveness to neglected health needs of vulnerable groups in Ghana and Vietnam, and (2) an empirically-grounded and theoretically-informed model of complex relations between the contexts, mechanisms and outcomes of the interventions, along with transferable best practices for scalability (i.e. expansion within similar contexts) and generalisability (i.e. to different contexts, such as other health areas and countries) for future health systems strengthening.

### Theoretical framework

The most widely known framework for health systems responsiveness, developed by the WHO in 2000, comprises seven domains: dignity, autonomy, confidentiality, prompt attention, quality of amenities, access to support networks, and choice of service provider [1, 8, 24]. The WHO framework has been translated into a survey toolkit for self-assessments of patients’ use of healthcare [24–26].

More recent work on the topic stressed that interactions between the people and their health systems are central to understanding this concept [2], and improving responsiveness should therefore address both the ‘people’ and the ‘systems’ sides of such interactions [2, 27, 28]. Evidence also shows that interpretations of responsiveness can be *context-sensitive* (e.g. expectations of dignity reflect political, democratic and policy climate [5]), and *vary across actors* (e.g. patients and providers with different powers [7, 29]) and *health facilities* (e.g. public/private [5, 7]). Responsiveness, therefore, is arguably a socially-constructed, rather than an ‘absolute’ and ‘universally normative’ concept.

Adaptations of the seven domains of responsiveness have been proposed for different health areas, such as HIV/AIDS or mental health [26, 30, 31]. Yet, we found no studies which sought to improve health systems responsiveness to health needs of vulnerable groups in LMICs, including to neglected maternal mental health needs. Furthermore, many theoretical and applied questions remain unanswered, such as the following. What does responsiveness mean to different actors on the ‘people’ and the ‘systems’ sides? How do these conceptualisations shape actors’ behaviours and practices? In what way improving responsiveness contributes to other health systems goals?

In RESPONSE, we will bridge some of these knowledge gaps while also responding to calls for improved understanding of health systems responsiveness to the needs of diverse health systems actors in LMICs [2, 11, 27, 28].

We approach ‘responsiveness’ as a dynamic social action which is produced via relationships between different actors within contexts of particular socio-economic arrangements as they negotiate experiences of professional, citizen, consumer and patient rights and responsibilities.

**Fig 2:**
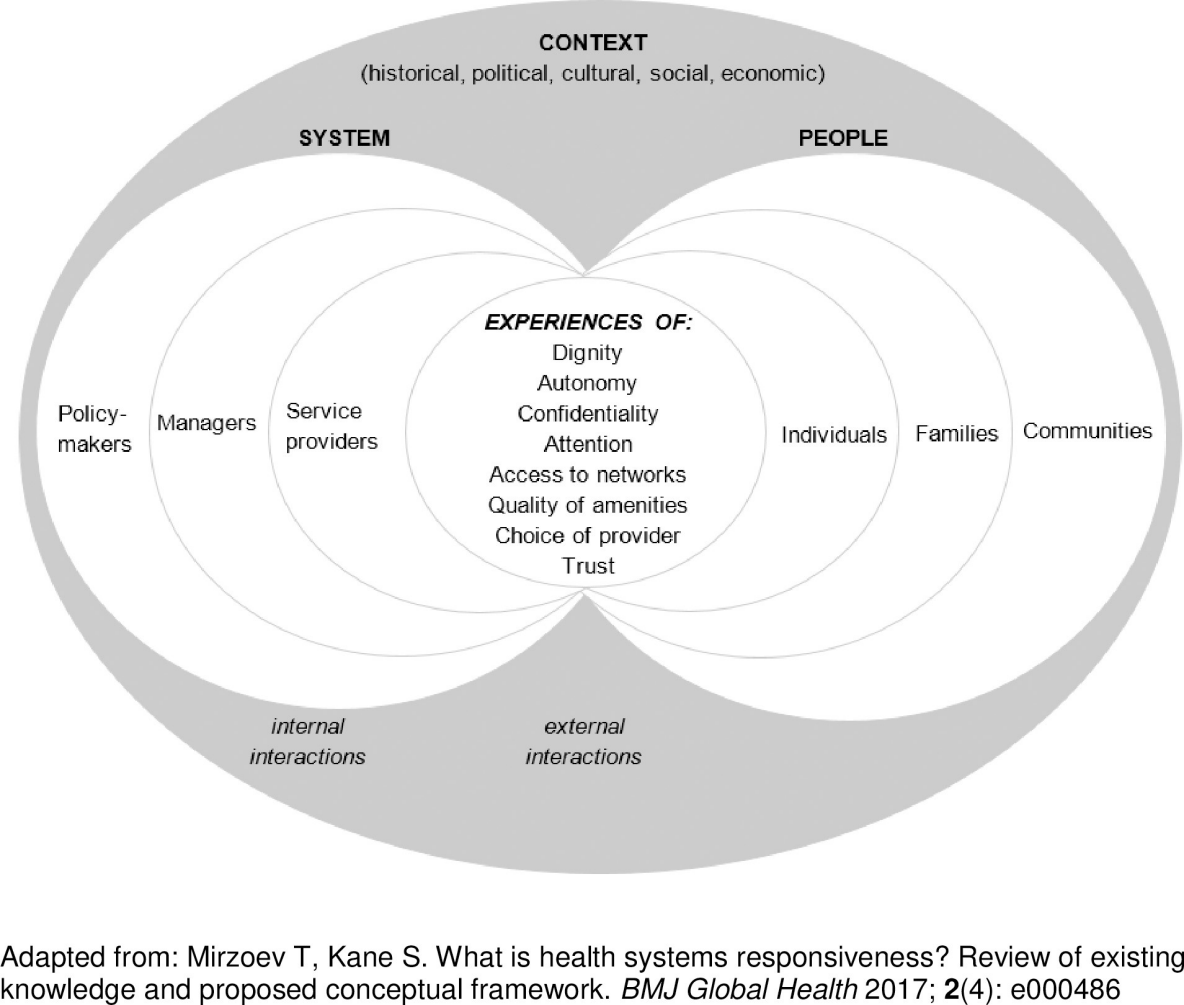
Framework for health systems responsiveness.

In our theoretical framework (Fig 2), health systems responsiveness is understood as comprising the socially-constructed processes of external and internal interactions. Experiences of these interactions determine the degree of health systems responsiveness across eight domains: dignity, autonomy, confidentiality, attention, access to networks, quality of amenities, choice of service provider and trust. People’s engagements with health systems (for example, to seek healthcare) and system’s responses to these engagements (for example, service delivery) are shaped by their initial expectations [2]. People’s initial expectations are influenced by their individual’s relations with their families and communities within the context of cultural and societal norms as well as their potential vulnerability. The system’s ability to be responsive is driven by the expectations of frontline service providers of responsive services, in the context of current structures, processes, priorities and targets set by policymakers and managers.

Conceptually, health systems responsiveness involves two socially-constructed interactions:

- *internal* i.e. between policymakers, managers and service providers, for example as part of prioritisation, resource allocation, target-setting, staff supervision and performance appraisal
- *external* i.e. between people and the system, typically during service provision [27, 28]

People’s interactions with their families and communities are conceptualised as a context which shapes their initial expectations and subsequent engagements with a health system, though these can also be interpreted as a possible third type of interactions.

Improving health systems responsiveness should, therefore, target both the internal (within the health systems) and external (between the people and health systems) interactions and be cognisant of the wider context which shapes people’s initial expectations of, and their subsequent engagements with, their health systems.

### Study setting

We will conduct the study in two lower-middle-income countries: Ghana and Vietnam. Our choice of these two countries is driven by three considerations. First, their *commonalities and diversity provide excellent value for this research*. Key similarities are:

- Both Ghana and Vietnam are rapidly transitioning LMIC contexts with stable political climate and socio-economic growth (Table 2), but with inequitable distribution of development gains [19, 32]. The fragile states index ranks them as 110^th^ and 109^th^, respectively (https://fragilestatesindex.org).
- In both countries, the most vulnerable are the poor and those have been left behind amidst major developmental gains for the rest of the country. In response, policymakers in each country are committed to the agenda of health equity and universal health coverage.
- Both countries have largely publicly funded national health systems, comprising four-tiered organisations with national, regional or province, district and local levels. Both health systems have a high degree of decentralization and a growing private sector.

**Table 2:** Key common indicators between Ghana and Vietnam.

Indicator	Ghana	Vietnam
OECD DAC Economy Classification	Lower Middle Income	
GNI per capita (2011 PPP$)	4,096	5,859
UNDP Human Development Category	Medium	Medium
Inequality-Adjusted Human Development Index (IHDI)	0.42	0.57
Socio-demographic Index (SDI)	0.53	0.6
Gender Development Index (GDI)	0.9	1.05
Healthcare Access and Quality (HAQ) Index	25.6 (22.5–28.9)	36.6 (33.1–40.4)
Antenatal care (ANC) coverage—at least four visits	87.3%	73%
Births attended by skilled birth attendants (SBAs)	71%	94%
Out of Pocket from the Total Health Expenditure	38%	45%

Data from

• Global Burden of Disease Collaborative Network. GBD Study 2017 Socio-Demographic Index (SDI) 1950–2017. Seattle, United States: Institute for Health Metrics and Evaluation (IHME); 2018.

• Fullman N, et al. Measuring performance on the Healthcare Access and Quality Index for 195 countries…: a systematic analysis from the GBD Study 2016. *The Lancet* 2018; **391**(10136): 2236–71.

• WHO. Global Health Observatory. http://apps.who.int/gho/portal/gho.jsp (accessed 9 May 2019).

• WHO. Global Health Expenditure Database. http://apps.who.int/nha (accessed 9 May 2019).

The two health systems also have differences. Vietnam’s publicly-dominated system has increasing market competition, but the Ghanaian system has large private not-for-profit sector and a more substantial share of external funding for health (13% compared with 2% in Vietnam) [33]. With regards to decentralisation, in Vietnam most power is consolidated at the province (regional) level whereas in Ghana districts have greater autonomy. While the antenatal care coverage and skilled birth attendance levels are relatively high (over 85%) in each country, maternal mortality in Ghana is higher i.e. 319/100,000 live births compared with 54/100,000 in Vietnam [34]. This may reflect problems with service quality and poorer people-system interactions leading to delays in seeking healthcare. In Ghana, there are dedicated staff for local mental healthcare (e.g. community psychiatric nurses), whereas provision of grassroots level mental healthcare in Vietnam is through mainstream primary health care workers. Our experience shows that in Vietnam, vulnerability often relates to ethnicity and income, but in Ghana it seems to be mostly location and income-related.

The second reason which guided the choice of Ghana and Vietnam is that there is *high interest from policymakers* in making the health systems more responsive to health needs of the most vulnerable. Preliminary discussions with key decision-makers from the Ghana Health Service and Ministries of Health in each country held during the proposal development showed that all key policymakers are committed to improving health systems responsiveness. In each country, maternal health remains a priority [35, 36] with increasing attention to mental health and integrated and responsive services. Psychological well-being of mothers and new-borns is a part of Ghana’s 2014 National Reproductive Health Policy and Standards. In Vietnam, policymakers emphasise ensuring equity in access, integrating mental healthcare (for example, into community programmes [37]) and piloting models of support and rehabilitation for those with mental illnesses.

Finally, we have chosen these two countries because *we have strong and longstanding academic collaborations* between the Universities of Leeds and Ghana [38], and Universities of Leeds and Melbourne with Hanoi University of Public Health [39, 40]. We will leverage and extend these into effective South-South collaborations, exchange and learning, as well as building links amongst policymakers and practitioners in Ghana and Vietnam.

In each country, we have purposefully selected one region (in Ghana) or province (in Vietnam) with some of the largest inequities disproportionately affecting vulnerable groups. Within each region and province, we have further selected a rural and an urban district to allow comparisons between rural-urban contexts.

In Ghana, we will work in Greater Accra Region located in the Coastal zone. It is 90% urban with highest maternal mortality of 336/100,000 live births as compared with the national average of 310/100,000 live births according to the 2017 Maternal Health Survey, growing squatter urban slums and some of the most deprived rural communities. In Vietnam, we will work in Bắc Giang, a mountainous Province 50km to the east of the capital Hanoi, with large vulnerable groups and 12% of the population comprising ethnic minorities.

In each country, we have selected two district health systems as our case studies, based on consultations with policymakers and their demographic and health indicators (Table 3) and leadership’s commitment to and opportunities for change. We have selected a purely rural (Shai Osudoku and Yên Thế), a rural with urbanised periphery (Ningo Prampram) and urban (Hiệp Hoà) districts, to allow learning across rural-urban settings.

**Table 3:** Key characteristics of study sites.

	Ghana			Vietnam		
	Shai-Osudoku	Ningo Prampram	Greater Accra Region	Yên The (rural and mountainous)	Hiep Hoà (urban)	Bac Giang Province
Total population	61,905	81,296	4,297,465	110,920	247,460	1,803,950
ANC 4+ visits	94.8%	98.2%	81.5	91.7%	99.27%	84.02%
Facility delivery rate	79.5%	81.7%	91.9	100%	99.93%	99.98%
Mental health	data not available					

Data from national health information systems

Both districts in Ghana have a Demographic Surveillance System run by the Dodowa Health Research Centre, thus giving a useful link to an existing dataset. The Vietnam’s MOH has identified the Bắc Giang Province to pilot models of support and rehabilitation for those with mental illnesses (including pregnant women), which provides an excellent opportunity and timing for our results to inform on-going reforms.

In each district, we will implement interventions in the district hospital, 2–4 public and private primary health care facilities and within respective communities. As we explain later in the paper, the interventions will aim to improve external interactions (i.e. between people and health systems) as well as internal interactions within health systems (i.e. between healthcare staff and managers). Simultaneously, in Ghana we will engage with Greater Accra Regional Health Directorate and Ghana Health Service, in Vietnam we will engage with Province Health Department, and national Ministry of Health. The project co-investigator Dr Ashinyo is Deputy Director in the Directorate of Clinical Care of the Ghana Health Service and therefore has direct links with national policymakers. In Vietnam, we will leverage project co-investigator Professor Bui’s strong research-policy links. This will ensure sustainability, replication and scaling up of the interventions.

### Study design

We will use a *mixed-methods realist theory-driven case study design*, utilising our expertise in realist evaluations [41–46] and established standards for reporting realist evaluations [43]. Health systems comprise heterogeneous interconnected actors (e.g. patients and providers) operating at multiple levels within a complex dynamic system. Ensuring systems responsiveness requires aligning multiple interpretations from actors with different powers and resources. A realist approach helps make sense of such complexity by identifying how the multiple components interact in non-linear ways [47, 48]. It recognises micro, meso and macro contexts (Cs) in triggering the mechanisms (Ms) to produce outcomes (Os) [47, 48], known as CMO configurations, to explore what works, in which circumstances, for whom and why [47], and therefore suits this study.

Realist researchers develop, test and refine middle-range theories which show causal pathways of how interventions work. Such an approach is similar to studies guided by theories of change or even initial hypotheses. Our draft initial theory, to be further developed, tested and refined is:

*Context-sensitive interventions for better recognition of initial expectations of key actors, if co-produced by these actors to target internal and external interactions and implemented within favourable policy contexts, will improve health systems responsiveness to neglected health needs of vulnerable groups, ultimately contributing to better health outcomes for all*.

**Fig 3:**
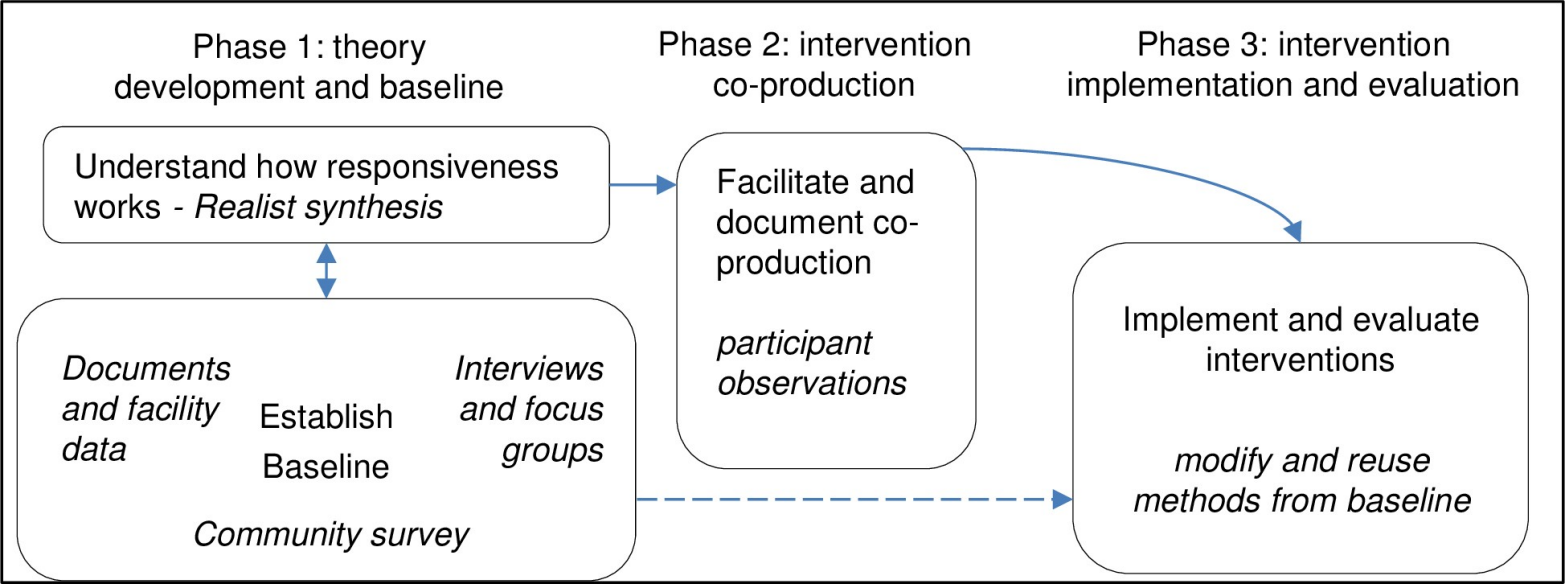
Project phases and methods.

This 42-months study will comprise three Phases as shown in Fig 3 (methods are in italics) and set out in detail in the following three sub-sections.

#### Phase 1: Theory development and baseline

In phase 1 (first year) we will understand actors’ expectations of responsive health systems, identify key priorities for fine-tuning interventions, develop initial theory (using evidence from a realist synthesis) and generate a baseline of data (through primary data collection and analysis). This phase will particularly address the study’s objective 1 and will include two broad parts:

- conducting realist synthesis using established processes [49, 50] to understand how responsiveness works and advance our initial working theory for its testing, validation and refining and
- collecting and analysing primary data using interviews, focus group discussions and community surveys, in order to understand actors’ expectations of responsive health systems, identify issues for fine-tuning the interventions in Phase 2 and establish a baseline for the interventions.

We will rigorously synthesise knowledge on systems responsiveness in LMICs using realist synthesis, a “…systematic, theory-driven interpretative technique… to help make sense of heterogeneous evidence about complex interventions applied in diverse contexts” [51 p.2]. This review approach is well suited for explaining disparate data and conceptualisations across the academic disciplines [52] and unpacking specific pathways of how particular contexts may trigger (or interfere with) mechanisms to produce intended or unintended outcomes [53]. The realist synthesis will help understand how specific micro-meso-macro contexts shape certain pathways through which responsiveness works. While there is a substantial knowledge base on assessing health systems responsiveness using adaptations of the WHO survey toolkit, published literature on improving systems responsiveness is scarce. Therefore, in addressing key issues from Phase 1 and fine-tuning the interventions (in Phase 2), we will draw on broader relevant knowledge of health systems strengthening–for example, accountability [6, 54, 55], integration of mental and maternal services [20, 22, 23] and staff support [56–58]–and will relate these to our understanding of vulnerability [14–17], our understanding of responsiveness in our framework and our initial theory.

Our realist synthesis will utilise a four-step process [59], which will run alongside other components of the wider RESPONSE study, and will comprise:

1. initial screening to map theoretical landscape of health systems responsiveness,
2. formulating initial programme theories on the basis of theorisation of health systems responsiveness
3. refining programme theories through purposeful screening of the literature and consultations with key stakeholders as part of the intervention co-production for improving health systems responsiveness in Ghana and Vietnam
4. testing programme theories through empirical evaluations of implemented interventions in Ghana and Vietnam and against the substantive theories underpinning health systems responsiveness.

A detailed protocol for the realist synthesis is available from the International Prospective Register of Systematic Reviews, PROSPERO registration CRD42020200353 (https://www.crd.york.ac.uk/prospero/display_record.php?RecordID=200353).

The aim of collecting and analysing primary data during Phase 1, will be to answer research questions 1a and 1b (see Table 1 earlier) through exploring:

- what responsiveness means to people across intersectionality dimensions (e.g. income, gender, social strata) and how this shapes their initial expectations from and interactions with, health systems within local contexts (e.g. through patient feedback [6, 60, 61]);
- how responsiveness is understood and enacted at different health systems levels (facility, district, regional, national) within the bureaucracies of health systems [27] and involving interactions between policymakers, managers and service providers [62];
- what health systems responsiveness means within preventive and curative settings of public and private healthcare facilities. We recognise, however, that public-private distinctions are increasingly blurred, with many private (not-for-profit) facilities sharing public-sector values and principles, and public-sector facilities facing market pressures.

We will collect data using: (i) reviews of policy and facility documentation including analysis of facility records of service provision and use; (ii) in-depth interviews and focus group discussions with key actors from local communities and health systems and (iii) a community survey. Data will be analysed retroductively, meaning both inductively and deductively. We will draw upon established processes for data analysis such as thematic analysis of qualitative data, and regression models for analysis of quantitative data.

We will begin with a comprehensive *review of policy and facility documentation* to deepen our understanding of policy, regulatory and systems environments and healthcare practices—relating all back to the conceptualisations of health systems responsiveness. We will qualitatively review two purposefully-identified types of documents: key national-level policies, plans and guidelines; and relevant local-level documents within health facilities, such as minutes of management meetings, clinical reviews, and staff management and performance appraisal records. We will adopt a semi-structured template to summarise insights on the underlying conceptualisations, values and ideas around responsiveness, and approaches to ensuring health systems responsiveness. We will also quantitatively assess patterns of provision and utilisation of maternal and mental health services. To do so, we will analyse facility records for the last 2–3 years disaggregated by key dimensions of intersectionality such as age, income and residence.

Using the *IDIs*, we will understand what responsiveness means to different health systems actors including its importance, underlying principles, components, mechanisms and intended outcomes. We will also explore the actors’ framing of underlying fundamental issues (e.g. rights, agency) and practices (management, service provision, health-seeking), relating them to our continuously refined understanding of responsiveness. From our experience, 20–25 IDIs should sufficiently represent views of key actor groups in each district, with further 10–15 IDIs at the province and national levels each (total 60–80 IDIs per country). This may decrease if we reach data saturation earlier, i.e. when no major new themes will be emerging from subsequent IDIs.

We will also conduct 4–6 *FGDs* with key actors in each country. These will explore their understanding and expectations of responsive health systems, their framing of underlying fundamental issues and will understand group norms and dynamics. The FGDs will be conducted at the community, facility and province levels (1–2 at each level). Each group will comprise 6–8 participants to maximise engagement, will comprise similar participants (in terms of age, staff category) and separate FGDs may be conducted to reduce gender-related bias.

The participants for IDIs and FGDs will be identified through purposive sampling and will include: (a) patients and public across their intersectionality dimensions (such as different genders, incomes and social strata); (b) health facility staff including service providers and support staff; and (c) health policymakers and managers. An initial participant list will be drawn by month 3 and we will identify further ones through snowballing. All FGDs and IDIs will be guided by a semi-structured topic guide to explore causal pathways of responsiveness [42]. Questions will be adapted to specific individuals’ backgrounds and roles. All IDIs and FGDs will be conducted in local languages as appropriate, audio-recorded, transcribed and either translated verbatim to English for thematic analysis or analysed in local languages with relevant extracts to be translated for cross-country comparisons.

We will conduct a baseline *community survey* to help us understand which attributes of responsive health systems community members value and expect most, and which drivers determine health-seeking behaviours of vulnerable groups. These attributes will be based on the WHO’s seven domains of health systems responsiveness: *dignity*, *autonomy*, *confidentiality*, *prompt attention*, *quality of amenities*, *access to support networks*, *and choice of service provider* [1, 8, 24]. We will explore how the values and expectations that respondents hold about these attributes, differ across key characteristics, such as users vs non-users of the health system, and across their different intersectionality dimensions, such as gender and socio-economic status. We will use insights from the preceding qualitative methods to guide the design of the survey questionnaire. We will measure how much relative importance respondents place on the different attributes of responsiveness, that is how much they value and expect each attribute, using Likert scales or discretely coded visual analogue scales. We will pilot test the questionnaire among a small number of purposively selected respondents to explore its acceptability and their understanding of a range of different scales, to allow us to select the most suitable scale. During the pilot we will also explore respondents’ understanding of the rest of the questionnaire, along with the feasibility and acceptability of the survey methodology to data collectors. We will also collect a range of key socio-demographic details from respondents on factors that are likely related to their access and use of the health system and their experiences of engaging with the health system, and which will allow us to categorise them in terms of key determinants of intersectionality like gender and socio-economic status.

Depending on the sampling frame data that is available in each country, we will use a multi-stage clustered household survey sampling approach [63] to allow us to select a statistically representative population sample from the relevant communities around our intervention facilities. *Sample size*: To understand respondents’ views on the importance of the different attributes of responsiveness, we will treat each discrete point on our chosen responsiveness attribute “importance scale” as a binary outcome and estimate the percentage of respondents selecting that point on the given scale (accounting for the complex survey design). We estimate that for the survey in each country we will require 562 respondents (assuming a response rate of 95%) to estimate the percentage of respondents selecting each point on the scale (assuming the most conservative percentage of 50% when estimating the sample size for a binary outcome based on precision) with a margin of error of ± 6 percentage points (95% confidence intervals), which we judged to be a suitable balance between precision and resources, and assuming a moderate design effect of 2 given we have no existing data (the mean design effect in the Ghana Demographic and Health Survey 2014 was 1.5). We will aim to understand the relationships between the socio-economic factors and respondents’ views on the relative importance of the different attributes of responsiveness, such as differences between men and women. In doing so, we will use appropriate multiple linear regression models that treat the “importance scale” outcomes as continuous variables (and which adjust for any complex survey design features such as clustering, stratification and weighting). Based on this approach, the above sample size (562) would also allow us to detect differences between binary subgroups, such as men and women (assuming a maximum between-subgroup size ratio of ≤6:1), of 0.65 or greater points in their responses to any of the “importance scale” questions with 80% power (based on two-sided hypothesis testing at the 5% significance level, assuming t-distributed data, a response rate of 95%, a design effect of 2).

The outcome of Phase 1 will be an initial theoretical model explaining how health systems responsiveness works. It will explain how different contexts shape and trigger the mechanisms through which health systems responsiveness is enacted (or not) by the different actors to produce the intended and unintended outcomes.

#### Phase 2: Intervention co-production

In Phase 2 (months 13–18) we will co-produce the context-sensitive interventions to improve health systems responsiveness, addressing study objective 2. The co-production will be through 2–3 meetings in each district involving key actors (communities, service providers, facility managers, regional/province and national-level actors). These meetings themselves can also be seen as interventions, and will therefore involve elements of capacity strengthening and knowledge uptake. The meetings will be led by district (or regional) health leadership and facilitated by researchers who will present evidence from Phase 1, document causal pathways of how the interventions are intended to work (i.e. refine initial theory) and using participant observations following a semi-structured template reflect on the co-production processes in terms of clarity, inclusivity, transparency and effectiveness.

In each country, the interventions will seek to improve two key components of responsiveness:

- internal interactions i.e. within and across facility, district, province and national levels
- external interactions i.e. between the health systems and vulnerable groups.

The rationale is that these two components, when combined, will ultimately contribute to improving people’s experiences across the different domains of health systems responsiveness specified in our theoretical framework. Our focus on the processes of interaction is also driven by our intention to enact systemic change rather than target specific individual domains in a discreet manner.

The overall design of the interventions is shown in Table 4.

**Table 4:** Two components of the interventions in Ghana and Vietnam.

Internal interactions	External interactions
HWFC workshops with health workers. Possible themes: • Perceptions among vulnerable groups about health workers and services • Understanding and improving specific domains of responsiveness • Learning from patient feedback for service quality improvement	Nominal group techniques with communities and health workers. Possible themes: • Improving patient feedback channels for communicating initial expectations • Enhancing utilisation of maternal and mental health services by vulnerable groups • Context sensitive strategies for empowering people to engage with their systems

During the intervention co-production meetings we will fine-tune this general design (i.e. finalise themes, refine facilitation guidance and produce required materials), to be informed by better understanding of people’s initial expectations of responsive health systems which inform their interactions with their health systems from Phase 1.

In fine-tuning the interventions, we will consolidate, adapt and extend our work in Health Workers for Change (HWFC) workshops [58, 64], Continuous Quality Improvement (CQI) [65, 66], patient feedback systems [61, 67] and acceptability of maternal healthcare by vulnerable groups [39, 40]. Relevant published and unpublished results will be presented during intervention co-production meetings as a possible ‘menu’ of interventions for considerations by the key stakeholders.

To improve internal interactions, in each district we will conduct sets of six thematic workshops, following the HWFC approach, comprising frontline health workers and managers in the district hospital and PHC facilities. These are participatory, interactive 2-hour workshops, moderated by a skilled facilitator–usually a social scientist with experience in moderating group discussions. The workshop series stems from Paulo Freire’s work on transformational learning [68] and aims to help staff surface and critically reflect on their experiences, strengths and constraints in delivering responsive and quality healthcare. From these reflections, staff are then encouraged and supported, to develop relevant and feasible local solutions [69]. The six workshops are usually titled: “why I am a health worker”; “how do our clients see us”; “women’s status in society”; “unmet needs”; “overcoming obstacles at work” and “solutions”. The manual for the HWFC series was developed from initial work in South Africa and then refined following experiences in other African contexts. During co-production, we will align the themes with our initial theory of systems responsiveness specifically targeting the internal (and external) interactions and adapt facilitation guidance as appropriate.

Experiences of using HFWC approach in Ghana and other countries, show them as an effective platform for institutionalising a process of Continuous Quality Improvement (CQI) in healthcare facilities [58, 64]. Evidence from Vietnam also shows that similar facilitated stakeholder groups can contribute towards improved health outcomes [70]. The CQI emphasises the process of systemic change underpinned by gradual optimisation and improvement and organisational learning; and that healthcare quality needs to be satisfied for both service users and providers [65, 66]. Both CQI and HWFC have been shown to improve communication within health facilities [58, 65] and thus focus on both the people and the systems sides of health systems responsiveness.

To improve external interactions, to establish group consensus during these workshops, we will use a Nominal Group Technique (NGT)–a structured, multistep, group consensus building technique which comprises 6 steps: (a) individual writing of ideas; (b) group review and feedback; and (c) discussion, clarification and evaluation of each idea (d) ranking ideas in order of their significance; (e) discussion of the preliminary vote; and (f) final individual voting on significance of each idea.

We will draw upon the NGT’s documented effects on consensus building, including in Ghana and in Vietnam [71, 72], to improve external interactions through empowering people to engage with their health systems. To raise people’s awareness of possible options, we will utilise our knowledge of improving patient feedback (as channels for people to convey their expectations from and reflections on, systems performance [61, 67]) and improving use of maternal healthcare [39, 40]. Evidence shows that effective interventions to improve feedback systems should target all three steps in the process: collection of feedback (e.g. raising patients’ awareness), analysis within facilities (e.g. improving staff skills), and acting on the information (e.g. integrating learning into service quality improvement) [67]. We will also draw upon the knowledge that use of available healthcare by vulnerable groups requires people’s cultural acceptance of these services [39]–and which can be improved through raising awareness about, and increasing confidence in, health workers [40].

The interventions are intentionally designed as low-cost activities to be embedded within the current structures and processes to ensure their sustainability, replication and scaling up. Although the HFWC workshops and NGT intend to improve internal and external interactions respectively, each is also likely to bridge the external-internal boundaries. We also anticipate that cumulatively these two intervention components will raise awareness and empower health workers and communities, and consequently will provide a sustainable platform for problem analysis and solution seeking. Such a platform will contribute to systemic improvements in responsiveness as a key attribute of health systems strengthening.

#### Phase 3: Intervention implementation and evaluation

In Phase 3 (months 19–42) we will implement and evaluate the interventions within local contexts. The interventions will be implemented for one full year and through existing systems’ structures and processes. Such duration should help embed the interventions within annual health planning and budgeting cycles and thus ensure their integration within routine practices and longer-term sustainability.

Using realist evaluation [47, 73] we will test and refine our initial theory and intended intervention pathways from Phases 1 and 2. These will be compared to the actual performance of the interventions in improving internal and external interactions. We will relate results to any changes in key domains of responsiveness from our theoretical framework (e.g. dignity), within the complex context of vulnerability and assessed against the baseline. We will repeat our community survey within the same areas and asking the same questions. See *Phase 1* section for proposed details of the survey design, questionnaire topics and format, and sample size/analysis. With the baseline and follow-up community surveys we can then explore whether respondents’ views on the relative importance of different attributes of responsiveness have changed subsequent to the intervention. This will be done using appropriate multiple linear regression models that adjust for the complex survey design, and also control for likely important confounding variables.

We will also assess the interventions’ feasibility and acceptability by key actors and processes within and between Ghana and Vietnam, utilising the UK Medical Research Council’s framework for process evaluation of complex interventions [74]. In doing so, we will modify, extend and reuse the Phase 1 methods.

Our intra- and cross-country comparative analyses will allow us to develop transferable best practices for other areas and LMICs which also experience similar socio-economic growth and face pressures to effectively address the needs of vulnerable groups. These best practices will be in a form of a theory-informed and empirically-grounded model of health systems responsiveness to neglected health needs of vulnerable groups, specifically addressing the study objectives 3 and 4. The model will guide a deeper understanding of how the contexts shape and trigger specific mechanisms through which health systems responsiveness works for different actors. We will also produce detailed practical guidance notes on further adaptations of this model to inform future policy and practice on improving health systems responsiveness in LMICs.

### Ethics and research governance

Ethics approvals for this study were obtained from the University of Leeds School of Medicine Research Ethics Committee (ref: MREC19-051), Ghana Health Service Ethics Review Committee (ref GHS-ERC 012/03/20) and Hanoi University of Public Health Institutional Review Board (ref 020-149/DD-YTCC). All primary data will be collected after obtaining written informed consent (or thumb print for those who can’t write) and while preserving participants’ anonymity and confidentiality.

The project will be carried out with full respect of current relevant legislation such as the General Data Protection Regulation. The methods development, data collection and analysis will consider:

- *anonymity* of all study respondents will be preserved where possible and will be ensured at all times if respondent(s) request. Unnecessary collection of personal data will be avoided. Where personal data is collected it will be coded, removed from the data for analysis and stored separately from transcripts. Only PIs and designated research personnel in each organisation will have access to the keys linking the data with the personal information.
- *informed consent* will be obtained from all study participants and in the case of refusal, alternative means of data collection will be explored (e.g. alternative respondents)
- specific emphasis will be placed on *confidentiality and other data protection issues*, which will include secure data storage and clear access rights. Only members of teams identified by the PIs in each institution will have access. Where data (e.g. transcripts) is stored on a server it will be password-protected and only research teams will have access to the passwords. Availability of documents on the internet will be following the consent of all collaborators.

The project will be implemented according to established robust research governance practice standards at the University of Leeds for implementing collaborative projects. This includes ensuring: regular communication between the partners and engagement with policymakers and practitioners; quality assurance through regular peer-review both within and between the teams; appropriate mentoring and coaching support to early career researchers; and equal opportunities as part of the Leeds University’s commitment to the Athena Swan equal opportunities initiative.

### Communication of project results

We will embed the research into policy and practice working with facility, district, regional and national actors, and through extending our academic collaborations into South-South research and policy partnerships. Engagements of decision-makers will facilitate implementation and scaling-up of these and similar interventions within and across Ghana and Vietnam.

We will communicate research findings through combinations of:

- developing policy briefs addressed to national and international policymakers and practitioners and designed as short and practical documents;
- delivering presentations at review meetings at local, district, regional/province and national levels in Ghana and Vietnam (e.g. quarterly, semi-annual and annual reviews involving national and international policy actors) and relevant regional meetings;
- regular (e.g. quarterly) project review meetings with national, regional and district actors and continuous engagement with key decision-makers through for example sharing draft policy implications for discussion of feasibility before including them in official reports;
- developing newsletters and press-releases aimed at communicating the key project findings in simple ways accessible to the general public in each country and their respective regions;
- interviews in the national media (e.g. radio and television) as well as articles for national newspapers, communicating our findings and educating the public on effective engagements and ensuring health systems responsiveness as appropriate and feasible;
- creating a website where all materials will be easily accessible by (inter)national decision-makers and practitioners, with mutual links to GHS and MOH websites in Ghana and Vietnam
- delivering presentations at national, regional and international conferences and publication of articles in peer-reviewed journals with specific emphasis on open access where feasible
- developing project reports for key stakeholders, with a publishable executive summary.

There is a high interest from key policymakers in this topic, some of whom are the members of the project team. We will maintain equal research-policy partnerships [75] and will embed research into policy and practice to facilitate integrating interventions within district health systems and ensure their sustainability.

## Discussion

This paper summarised the protocol for a mixed methods realist evaluation study which aims to understand and improve health systems responsiveness to health needs of vulnerable groups in Ghana and Vietnam.

A key study’s theoretical outcome will be an empirically-grounded and theory-informed model of complex contexts-mechanisms-outcomes relations, together with transferable best practices for scalability (i.e. expanding within similar contexts) and generalisability (i.e. expanding to other contexts e.g. other health areas and countries) for future health systems strengthening. The model will advance the current understanding of health systems responsiveness and will form an overall theoretical contribution from this study to the published knowledge on the subject.

Decision-makers from facility, district, regional and national levels will be engaged through embedded research in policy and practice and equal research-policy partnerships [75]. This will ensure the ownership and buy-in from the key stakeholders throughout the study and sustainability of project impacts in the longer-term. The economic and societal impacts will be generated through improvements in: health systems responsiveness, health policy implementation and health service provision, health systems performance and ultimately health outcomes and equity.

Capacity strengthening is a distinct project objective and is therefore an important aspect of this study. We will deploy a systematic approach to capacity strengthening which will be underpinned by our in-depth understanding of current capacity needs and assets of each partner team from previous studies [38, 40, 61, 76], and will comprehensively target individual, organisational and system levels of capacity. At the *individual level* we will seek to improve knowledge and skills of early-career researchers particularly in Ghana and Vietnam through mentoring support and on-the-job training (e.g. to lead academic publications). We also aim to improve individual capacities of key decision-makers to effectively set research priorities and understand and apply research results in their routine practices, through their continuous engagements in the study. At the *organisational level*, we will improve partners’ processes for research governance (e.g. finance management, reporting) and their ability to effectively communicate results. *Systems level* work will target partners’ networks including strengthening and expanding existing research-policy links in Ghana and Vietnam. During the first three months of the project, we will consolidate our in-depth knowledge of partners’ current capacities and will develop a clear, coherent and feasible capacity strengthening plan. The South-South exchange and learning will be central to our capacity strengthening approach with collaborators in Ghana and Vietnam owning and driving capacity strengthening. Senior co-Is in Ghana and Vietnam will mentor early-career researchers and create organisational environment where researchers’ enhanced expertise can be effectively applied.
